# Contribution of inter-muscular synchronization in the modulation of tremor intensity in Parkinson’s disease

**DOI:** 10.1186/s12984-015-0101-x

**Published:** 2015-12-01

**Authors:** Xin He, Man-Zhao Hao, Ming Wei, Qin Xiao, Ning Lan

**Affiliations:** Institute of Rehabilitation Engineering, Med-X Research Institute and School of Biomedical Engineering, Shanghai Jiao Tong University, 1954 Hua Shan Road, Shanghai, 200030 China; Department of Neurology & Institute of Neurology, Ruijin Hospital, School of Medicine, Shanghai Jiao Tong University, Shanghai, 200025 China; Division of Biokinesiology and Physical Therapy, Herman Ostrow School of Dentistry, University of Southern California, Los Angeles, CA 90089 USA

**Keywords:** Parkinson’s disease, Resting tremor, Inter-muscular synchronization, Electromyography, Correlation analysis, Antagonistic muscles

## Abstract

**Background:**

Involuntary central oscillations at single and double tremor frequencies drive the peripheral neuromechanical system of muscles and joints to cause tremor in Parkinson’s disease (PD). The central signal of double tremor frequency was found to correlate more directly to individual muscle EMGs (Timmermann et al. 2003). This study is aimed at investigating what central components of oscillation contribute to inter-muscular synchronization in a group of upper extremity muscles during tremor in PD patients.

**Methods:**

11 idiopathic, tremor dominant PD subjects participated in this study. Joint kinematics during tremor in the upper extremity was recorded along with EMGs of six upper arm muscles using a novel experimental apparatus. The apparatus provided support for the upper extremity on a horizontal surface with reduced friction, so that resting tremor in the arm can be recorded with a MotionMonitor II system. In each subject, the frequencies of rhythmic firings in upper arm muscles were determined using spectral analysis. Paired and pool-averaged coherence analyses of EMGs for the group of muscles were performed to correlate the level of inter-muscular synchronization to tremor amplitudes at shoulder and elbow. The phase shift between synchronized antagonistic muscle pairs was calculated to aid coherence analysis in the muscle pool.

**Results:**

Recorded EMG revealed that rhythmic firings were present in most recorded muscles, which were either synchronized to form phase-locked bursting cycles at a subject specific frequency, or unsynchronized with a random phase distribution. Paired coherence showed a stronger synchronization among a subset of recorded arm muscles at tremor frequency than that at double tremor frequency. Furthermore, the number of synchronized muscles in the arm was positively correlated to tremor amplitudes at elbow and shoulder. Pool-averaged coherence at tremor frequency also showed a better correlation with the amplitude of resting tremor than that of double tremor frequency, indicating that the neuromechanical coupling in peripheral neuromuscular system was stronger at tremor frequency.

**Conclusions:**

Both paired and pool-averaged coherences are more consistent indexes to correlate to tremor intensity in a group of upper extremity muscles of PD patients. The central drive at tremor frequency contributes mainly to synchronize peripheral muscles in the modulation of tremor intensity.

## Background

About 70 % of patients with Parkinson’s disease (PD) manifest conspicuous tremor at rest and/or during maintenance of posture [1–5]. The tremor dominant patients suffer milder rigidity and bradykinesia [6–9] with less impairment of motor functions [10, 11]. But the appearance of tremor significantly affects patient’s daily functions and social interactions.

The origin of resting tremor has been attributed to involuntary central oscillations in the central nervous system [12, 13]. Neuroimaging analysis indicated that the basal ganglia and cerebello-thalamo-cortical circuits are intimately involved in tremor generation [14, 15]. Within these two circuits, oscillations at tremor frequency have been observed from both deep brain structures [16–22] and cortical areas [14, 23]. Furthermore, oscillation at double tremor frequency detected from the primary motor cortex, which is the common output port of both circuits, was found to have a stronger coupling with muscle EMGs than that at single tremor frequency [14]. The double tremor frequency presented in central oscillations was not a harmonic component of the single tremor frequency in the signal, but each arose from different cortical and subcortical areas [14, 24–26]. In a previous study [27], we proposed a corticospinal model of tremor signal transmission based on the propriospinal neuronal (PN) network [28]. The PN network integrates the cortical oscillations at single and double tremor frequencies and divides them into two alternating activation bursts to drive a pair of antagonistic muscles respectively [27]. It is not yet clear to what extent a group of muscles in the upper extremity is recruited to participate in tremor generation.

Previous EMG studies revealed that different body parts exhibited uniform or different tremor frequencies [29, 30]. Coherence analysis further showed that tremor activities in different limbs were not phase locked, indicating that independent oscillators were involved in the tremor in different limbs [31–33]. However, significant coherence between muscles in one limb was detected in 70 % of tremor-dominant PD patients [32]. A recent study of the upper extremity under postural condition found a significant coherence in 4 of the wrist and elbow muscles in PD subjects [34]. The evidence appeared to imply that the central involuntary oscillations are modularized to affect a specific group of muscles in one limb, which synchronizes these muscles to contribute in tremor activity.

The objective here is to investigate how inter-muscular synchronization in a group of upper extremity muscles is correlated to tremor intensity. In this study, we developed an experimental method to quantify the neuromechanics of tremor in the arm and the synchronous EMG activities in a set of arm muscles in 11 tremor dominant PD patients. Frequency and coherence analyses (paired, pooled and pool-averaged coherence) were performed to evaluate inter-muscular synchronization among the group of muscles during tremor. Correlation between inter-muscular synchronization and tremor amplitude in joints was also assessed. Results found that inter-muscular synchronization in the upper extremity muscles contributes to modulate tremor intensity in PD patients. Further implication of these results is discussed with regard to how central oscillations at tremor and double tremor frequencies may be responsible for recruiting and driving peripheral muscles during tremor generation. Preliminary study of the experimental method validation was reported in a conference proceeding [35].

## Methods

### Subjects

Eleven idiopathic PD (Parkinson’s disease) subjects with tremor dominant symptoms manifest in upper extremity, with UPDRS (Unified Parkinson’s Disease Rating Scale) tremor subscore (item 16 + 20 + 21) of 6.2 ± 71.28, were recruited from the Department of Neurology, Ruijin Hospital (affiliated to School of Medicine, Shanghai Jiao Tong University). The tremor behaviors of the more affected side of upper extremity were recorded for elaborated tremor evaluation. The Ethics Committee of Animal and Human Subject Studies of Med-X Research Institute, Shanghai Jiao Tong University, approved this study. All the subjects signed informed consent before participating in this study. Information of the 11 PD subjects is listed in Table 1, including gender, age, and the test side of upper extremity. Additional information for each subject, such as UPDRS part III (clinical motor evaluation), H-Y (Hoehn and Yahr) Scale, and medication treatment as evaluated at the time of participation in the study is also given in Table 1.

**Table 1:** Clinical information of PD subjects recruited in this study

PD Subjects	P1	P2	P3	P4	P5	P6	P7	P8	P9	P10	P11
**Gender**	F	F	M	F	M	F	M	F	M	M	M
**Test Side ** ^a^	R	R	L	R	R	L	L	L	L	L	R
**Age (** ***yrs.*** **)**	60	62	63	65	65	56	63	59	80	65	76
**Disease Course (yrs.)**	6	5	6	2	15	1/2	3	10	10	10	6
**UPDRS Part III ** ^b^	16	22	16	25	32	15	17	16	24	24	17
**H-Y Scale ** ^c^	2	1	1	1.5	3	2	2	2.5	2	2.5	2.5
**L-Dopa Equivalents (mg/d)**	300.8	101.25	831.25	101.25	738.3	0	150	0	550.8	375	575.2

^**a**^Test Side was chosen by tremor originated side of PD subjects

^**b**^Unified Parkinson’s Disease Rating Scale, Part III: clinician-scored monitored motor evaluation (0 ~ 56)

^**c**^Hoehn and Yahr scale (1 ~ 5)

### Experimental setup

#### Movement platform designed for antigravity support

**Fig. 1:**
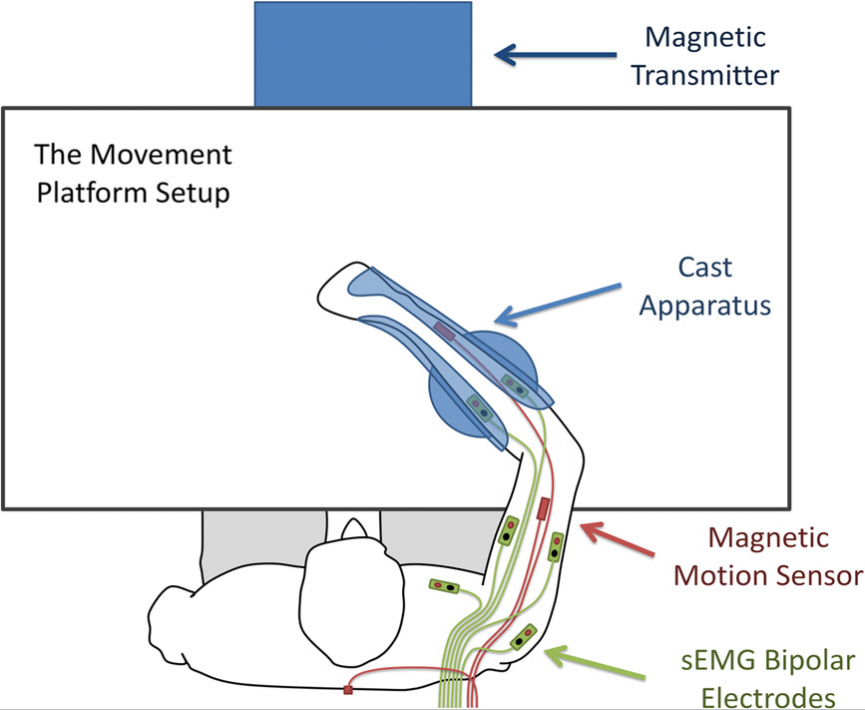
The experimental setup for Parkinsonian tremor recording. Six channels of surface electromyography (sEMG) were recorded from each subject by placing bipolar sEMG electrodes on top of muscles that exhibited evident involuntary oscillatory activities in the upper extremity. Joint movements were tracked by magnetic motion sensors attached to body segments in a gradient magnetic field generated by the magnetic transmitter. The cast apparatus with magnetic compatible design can provide antigravity support for the arm without affecting tremor behaviors by introducing significant damping and friction effects

To investigate resting tremor behaviors in the upper extremities of PD subjects with antigravity support, a motion platform and a fiberglass cast apparatus were custom designed to support the arm in the horizontal plane. The experimental setup is illustrated in Fig. 1. The subject sat comfortably at the table wearing a fiberglass cast apparatus on the forearm, and the height of the table was adjustable to suit the subject. The cast apparatus was designed with low-inertia, friction-free and magnetic compatibility consideration: the cast apparatus was assembled from a lightweight forearm shaped fiberglass cast and a plexiglass brace by nylon screws and nuts; 5 ball-bearing wheels made of silicon nitride (Si3N4) ceramic balls were mounted on the brace to support the forearm sliding on the platform. The cast apparatus wrapped and fixed the wrist joint to support the hand and to avoid hand dragging on the platform. The ergonomically designed cast apparatus was capable of supporting the upper extremity of subjects resting on the table effortlessly and moving in the horizontal plane easily. The motion platform and the cast apparatus were constructed without using any metal parts in order to work compatible with a magnetic motion tracking system. This setup for PD tremor evaluation was validated in a previous conference proceeding paper [35].

#### Surface EMG and kinematic measurements

Six channels of surface electromyography (sEMG) were collected from the upper extremity of the test side of each PD subject during planar postural task performance (Fig. 1). Six muscles that exhibited the most prominent oscillatory activities on the oscilloscopes were selected based on visual inspection from the following eight muscles to be recorded from each subject: flexor digitorum superficialis (FDS), extensor digitorum (ED), flexor carpi radialis (FCR), extensor carpi radialis (ECR), flexor carpi ulnaris (FCU), biceps brachii (Biceps), triceps brachii (Triceps) and deltoid anterior (DA). Recorded muscles of each subject are listed in Table 2. Surface EMG signals were recorded using Norotrode™ silver/silver chloride (Ag/AgCl) bipolar electrodes (Model BS-24SAF) and a copper pad reference [36]. The EMG signals were pre-amplified by 5000 times and band-pass filtered between 1 and 1000 *Hz* using Grass™ amplifiers. Then the EMG signals were A/D converted at a sampling rate of 2410 *Hz* using a Computing Measurement™ USB-BNC A/D card.

**Table 2:** Degrees of muscle synchronization determined by coherence analysis

PD Subject	Finger Muscles		Wrist Muscles			Elbow & Shoulder Muscles			Number of Synchronized Muscles
	FDS	ED	FCR	ECR	FCU	Biceps	Triceps	DA	
**P1**	**+**	**+**	**+**	**+**		**+**	**+**		**6**
**P2**	**+**	**+**	**+**	**+**		**+**	**+**		**6**
**P3**	**+**	**−**			**+**	**+**	**−**	**−**	**3**
**P4**	**+**	**+**			**+**	**+**	**+**	**−**	**5**
**P5**	**+**	**−**			**+**	**−**	**−**	**−**	**2**
**P6**	**+**	**+**	**+**	**+**		**+**	**+**		**6**
**P7**	**+**	**+**			**+**	**−**	**−**	**−**	**3**
**P8**	**+**	**−**		**+**	**+**	**+**	**−**		**4**
**P9**	**+**	**+**	**+**	**−**		**+**	**+**		**5**
**P10**	**+**	**−**	**+**	**+**		**−**	**−**		**3**
**P11**	**+**	**+**	**+**	**+**		**+**	**+**		**6**

Recorded muscles of each PD subject are denoted by ‘**+**’ or ‘**−**’ signs

‘**+**’ sign indicates the corresponding muscle is synchronized with all other ‘**+**’ muscles

‘**−**’ sign indicates the corresponding muscle is not synchronized with all ‘**+**’ muscles

A MotionMonitor™ II system (Innovative Sports Training, Inc. Chicago, IL, USA) was employed for movement recording. An Ascension™ wide range magnetic transmitter placed in front of the subject at a distance of 1.5 m generated a sphere gradient magnetic field with an effective radius of 3 *m*. Three magnetic motion sensors (Ascension™ trakSTAR, Model 800) were attached to 3 body segments (forearm, humerus, and thorax) respectively. Each sensor measured 6 signals, corresponding to the 6 DOFs (degrees of freedom) motion of a rigid body: 3 DOFs in Cartesian coordinates (spatial resolution: about 1 *mm*) and 3 DOFs in rotational coordinates (angular resolution: about 0.1°). The kinematic signals were first collected at 120 *Hz*, and then linearly interpolated to align to the EMG sampling rate (2410 *Hz*) for synchronized recording. Since the wrist joint was fixed by wearing the cast apparatus on the forearm, the other four channels of joint angle signals (shoulder flexion, shoulder abduction, shoulder rotation, and elbow flexion) were calculated from the raw sensor signals by algorithms implemented in the software of the MotionMonitor II system.

### Experimental procedure

PD subjects were instructed to rest their upper extremity on the platform, configured as illustrated in Fig. 1, with their hands positioned 30-50 *cm* in front of their chest. The subjects were instructed to count down from 100 vocally for distraction while resting tremor trials were recorded. Each trial ranged from 10 ~ 40 seconds, with 2 minute resting intervals between trials. Four trials that demonstrated conspicuous tremor activity in kinematic or EMG signals were recorded from each subject.

### Signal processing and data analysis

The collected data were processed and analyzed offline by custom developed Matlab programs (Version: R2010a, MathWorks Inc.).

#### EMG and kinematic signal pre-processing

**Fig. 2:**
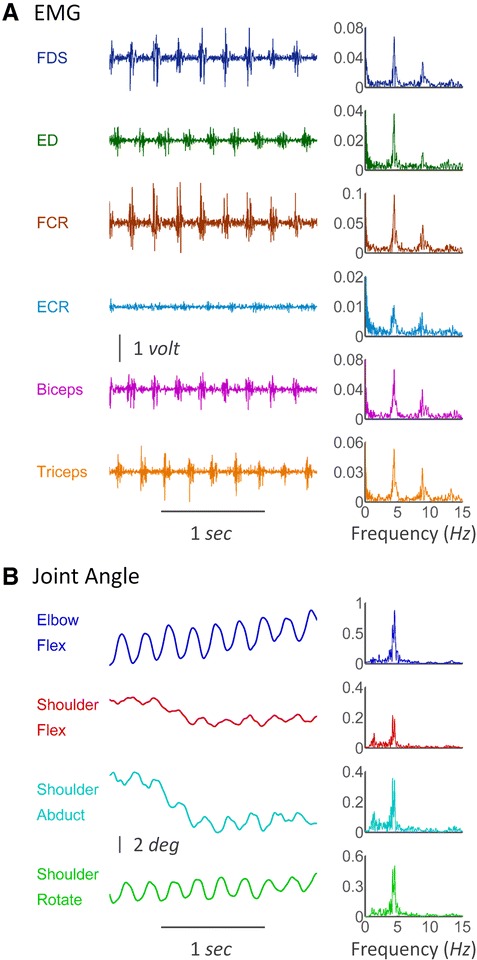
The sEMG and joint angle trajectory during Parkinsonian tremor recorded from PD subject P9. **a** The sEMG of 6 muscles are shown in the left column, and the corresponding spectra are shown in the right column. The EMGs show similar rhythms of spontaneous oscillatory activities across all muscles, and all the spectra show similar two major components at single and double tremor frequencies. **b** Oscillations in elbow (flexion) and shoulder (flexion, abduction, and rotation) joints are demonstrated in the left column, and the corresponding spectra are presented in the right column. Joint trajectory spectra show a single component at the tremor frequency

The raw EMG and motion signals were pre-processed according to the following steps to remove noise. Filters were implemented in both forward and backward directions to avoid phase distortions. The raw EMG signals were first notch filtered to remove the power line noise at 50 *Hz* and its higher harmonics up to 350 *Hz* (notch filter width 1 *Hz*, 14^th^ order Butterworth), as well as the magnetic noise produced by the magnetic transmitter of MotionMonitor II system at 120 *Hz* and its higher harmonics up to 360 *Hz* (notch filter width 1 *Hz*, 14^th^ order Butterworth). Then EMG signals were band-pass filtered with cut-off frequencies from 20 to 380 *Hz* (4^th^ order Butterworth) to remove high-frequency noise and low-frequency motion artifacts (Fig. 2a left column). The band-pass filtered EMG signals were rectified and then high-pass filtered with a cut-off frequency of 1 *Hz* (4^th^ order Butterworth) to remove the DC component for frequency analysis (Fig. 2a right column) and coherence analysis. The rectified EMG bursting patterns were low-pass filtered with a cut-off frequency of 7 *Hz* (10^th^ order Chebyshev type 1) to remove component at double tremor frequency for time delay and phase shift calculation.

The raw kinematic data of joint trajectories were low-pass filtered with a cut-off frequency of 20 *Hz* (16^th^ order Butterworth) to remove high-frequency noise (Fig. 2b left column). The de-noised trajectories were then high-pass filtered with a cut-off frequency of 1 *Hz* (4^th^ order Butterworth) to remove the DC component for frequency analysis (Fig. 2b right column) and tremor amplitude calculation.

#### Tremor frequency and amplitude calculation

The EMG tremor frequency of each muscle and tremor amplitudes were identified from several 1 *sec* epochs cut out from each trial by rectangular window, which exhibited evident tremor activities, and the frequency spectra of each epoch was calculated by the Fast Fourier transformation (FFT) algorithm. A frequency peak between 3 - 7 *Hz* was identified from each spectrum by the criterion that the peak amplitude reaches to a threshold at twice of the average of the corresponding spectrum between 3 - 7 *Hz*. The muscles with identifiable frequency peaks were labeled as rhythmic muscles, and those with no identified frequency peak in all epochs were labeled as non-rhythmic muscles. Therefore, for each rhythmic muscle, one tremor frequency could be detected from one epoch, and in total at least 10 tremor epochs with identifiable frequency peaks were identified from each subject. The EMG tremor frequency of each muscle was averaged from all tremor epochs, and one-way ANOVA (analysis of variance) tests were conducted to detect any difference in EMG tremor frequencies among different muscles within each subject. The characteristic subject-specific tremor frequency of each subject was calculated by averaging the EMG tremor frequencies of all muscles.

The amplitude of tremor in joints within each epoch was calculated from the joint trajectories with the DC component removed. Tremor amplitude of each joint DOF was defined as the range between the lower and upper limit of 95 % confidence intervals (determined by 1.96σ) of the corresponding joint angle distribution.

#### Degree of inter-muscular synchronization quantified by coherence analysis

**Fig. 3:**
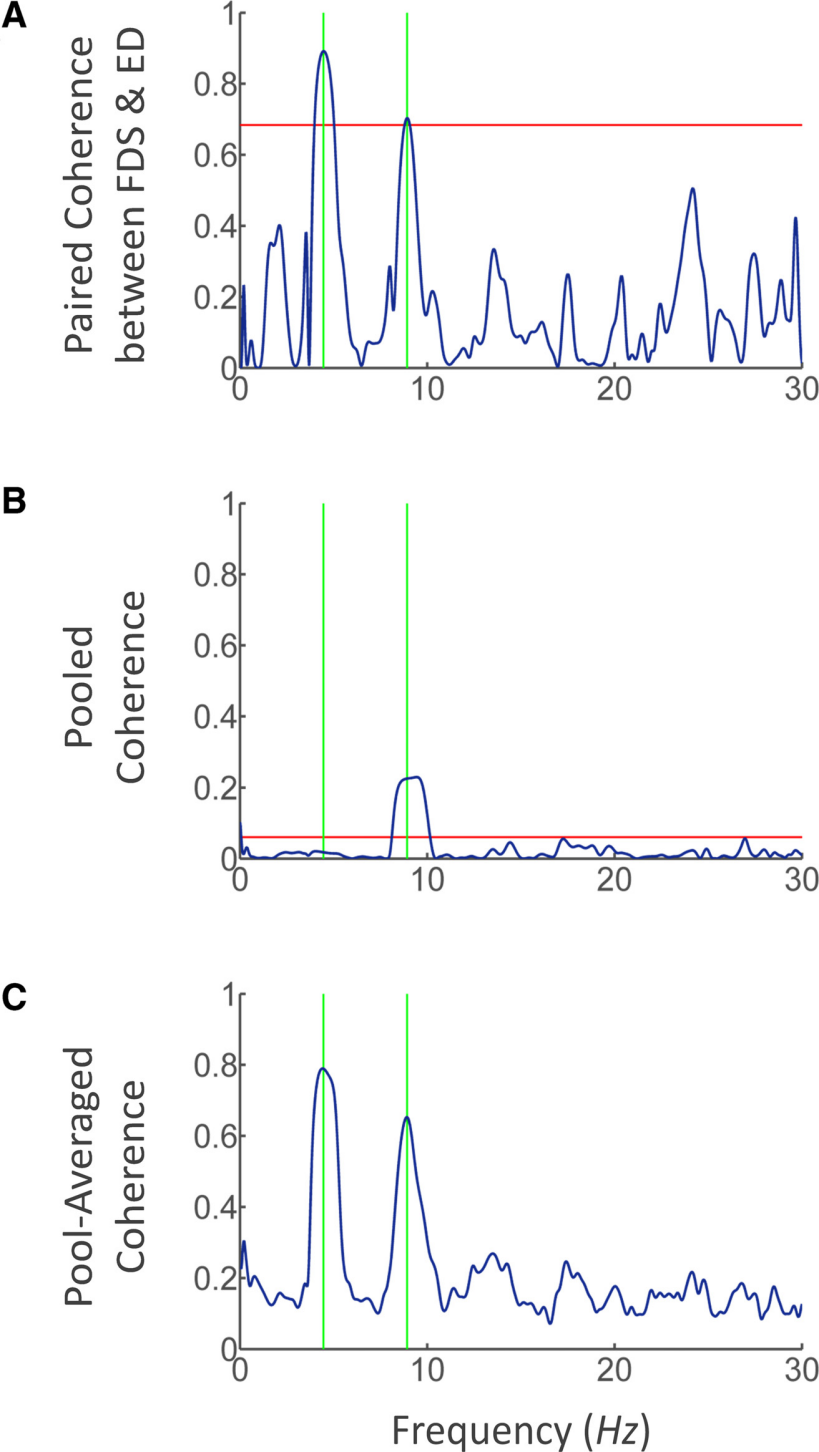
Coherence analyses for evaluation of inter-muscular synchronization. **a** The paired coherence between muscles FDS and ED in subject P9. The horizontal red line indicates the 99 % upper confidence limit of significant coherence level. The vertical green lines indicate the subject-specific tremor frequency and double tremor frequency of subject P9. **b** The pooled coherence of all 15 pairs of muscles among 6 recorded muscles in subject P9. The horizontal red line indicates the 99 % upper confidence limit of significant coherence level. **c** The pool-averaged coherence of all 15 pairs among 6 recorded muscles in subject P9

Three different modes of coherence analysis: paired coherence between muscles, pooled coherence of all muscle pairs, and pool-averaged coherence of all muscle pairs, were introduced to evaluate the level of inter-muscular synchronization of each PD subject. The magnitude squared coherence matrix ***M*** [37–39] comprising the coherence of all muscle pairs were calculated using Welch PSD (power spectral density) method [40, 41]:

1 $$ \begin{array}{l}{M}_{ij}\left(\lambda \right)=\frac{{\left|{S}_{ij}\left(\lambda \right)\right|}^2}{S_{ii}\left(\lambda \right)\cdot {S}_{jj}\left(\lambda \right)}\\ {}\left(i,\;j=1,\;2,\dots, 6\right)\end{array} $$

in which the cross spectral density *S*_*ij*_ and auto-spectral density *S*_*ii*_ at frequency *λ* were calculated from *L*(*L* = 5) segments of signals segmented by rectangular windows with length of *T* (*T* = 2 *sec*):

2 $$ {S}_{ij}\left(\lambda \right)=\frac{1}{2\pi LT}{\displaystyle \sum_{l=1}^L{F}_i\left(\lambda, \kern0.5em l\right)\cdot {F}_j^{*}}\left(\lambda, \kern0.5em l\right) $$

in which *F*_*i*_(*λ*, *l*) is the Fourier transform of segment *l* from EMG signal *i*, and F^*^ denotes the complex conjugate of *F*. Since the tremor activity occurred transiently during the recording, the number of segments *L* was determined by the subject with the least number of 2-sec tremor episodes that revealed prominent and stable tremor activities. The range of coherence was bounded between 0 and 1, and *M*_*ij*_(*λ*) = 1 indicated a perfect linear relation between EMG signals *s*_*i*_(*t*) and *s*_*j*_(*t*) at frequency *λ*. The upper 99 % confidence limit for significant coherency was determined by [38]:

3 $$ 1-{\left(1-\alpha \right)}^{1/\left(L-1\right)} $$

where *α* = 99% resulted in a coherence threshold of 0.68, which was adopted as a criterion to determine if the activities of a pair of muscles were strongly synchronized at the tremor frequency (Fig. 3a). The “tremor frequency” represents the “subject-specific tremor frequency” in here and the following text if no specific modifier is set before it.

The pooled coherence *P* [42] of all 15 pairs of muscles of each subject was calculated by:

4 $$ P=\frac{{\left|{\displaystyle {\sum}_{k=1}^{15}{S}_{a_k{b}_k}\left(\lambda \right)}\right|}^2}{\left({\displaystyle {\sum}_{k=1}^{15}{S}_{a_k{a}_k}\left(\lambda \right)}\right)\cdot \left({\displaystyle {\sum}_{k=1}^{15}{S}_{b_k{b}_k}\left(\lambda \right)}\right)} $$

where ${S}_{a_k{b}_k}\left(\lambda \right)$ denotes the cross spectral density of the k^th^ pair of signals a and b, estimated from *L*_*k*_(*L*_*k*_ = 5) disjoint segments. And the upper 99 % confidence limit for the pooled coherence estimation is given by [42] (Fig. 3b):

5 $$ 1-{(0.01)}^{1/\left({\displaystyle \sum {L}_k-1}\right)} $$

The pool-averaged coherence *PAC* of all 15 pairs of muscles of each subject is defined as:

6 $$ PAC=\frac{{\displaystyle {\sum}_{k=1}^{15}{M}_{a_k{b}_k}\left(\lambda \right)}\cdot {L}_k}{{\displaystyle {\sum}_{k=1}^{15}{L}_k}} $$

where ${M}_{a_k{b}_k}\left(\lambda \right)$ denotes the magnitude squared coherence of the k^th^ pair of signals a and b (*a* ≠ *b*), estimated from *L*_*k*_(*L*_*k*_ = 5) disjoint segments. Actually ${M}_{a_k{b}_k}$ (*k* = 1, 2, …, 15) are the 15 elements in the matrix ***M*** (magnitude squared coherence matrix), as defined by eq. (1), above the main diagonal elements.

The degree of inter-muscular synchronization of each PD subject was quantified by 3 different estimations: (1) the number of synchronized muscles, (2) the pooled coherence at tremor frequency and double tremor frequency (Fig. 3b), (3) the pool-averaged coherence at tremor frequency and double tremor frequency (Fig. 3c). As exemplified in Fig. 3a, muscles FDS and ED of subject P9 were identified as synchronized by paired coherence analysis, for which the coherence exceeded the 0.68 threshold at tremor frequency. The number of synchronized muscles was defined as the size of the largest sub-group of the recorded muscles, in which all muscle pairs were identified as synchronized by paired coherence analysis (Table 2).

#### Phase shift detected by cross-correlation

The time delay and phase shift between synchronized antagonistic muscles were determined by cross correlation analysis. Cross-correlation *XC*(*τ*) between 1-sec EMG epochs *s*_*i*_(*t*) and *s*_*j*_(*t*) of antagonistic muscles was calculated by:

7 $$ X{C}_{ij}\left(\tau \right)={\displaystyle \underset{-\infty }{\overset{+\infty }{\int }}{s}_i(t){s}_j\left(t+\tau \right)dt} $$

where τ is the lag, and the double tremor frequency component has been removed from the EMG epochs before cross-correlation calculation. Therefore, *τ*_0_ corresponding to maximum of *XC*_*ij*_(*τ*_0_) denotes the deviation of the major peak from the center of cross-correlation, which determines the time delays between shifted bursting patterns of antagonistic muscles. The phase shift φ between antagonistic muscles was calculated by:

8 $$ \varphi ={\tau}_0\cdot {F}_{tremor} $$

where *F*_*tremor*_ denotes the characteristic tremor frequency of each subject. Then the phase shift was normalized into one cycle from -60° to 300°. The phase shifts detected from all 1-sec epochs were averaged to gain the overall phase shift between antagonistic muscle pairs.

## Results

### Pathological rhythmic bursting in muscles

Most recorded muscles of the PD subjects demonstrated conspicuous rhythmic bursting activities during tremor, as exemplified in Fig. 2a, with a highly regular and rhythmic pattern. The EMGs of muscles in Fig. 2a shared a similar spectral density distribution with two major components, a larger peak at tremor frequency and a smaller peak at double tremor frequency. However, the spectra of joint kinematic signals in Fig. 2b contained only one major component at tremor frequency. The EMG bursts in antagonistic muscles are alternatingly organized at tremor frequency.

**Fig. 4:**
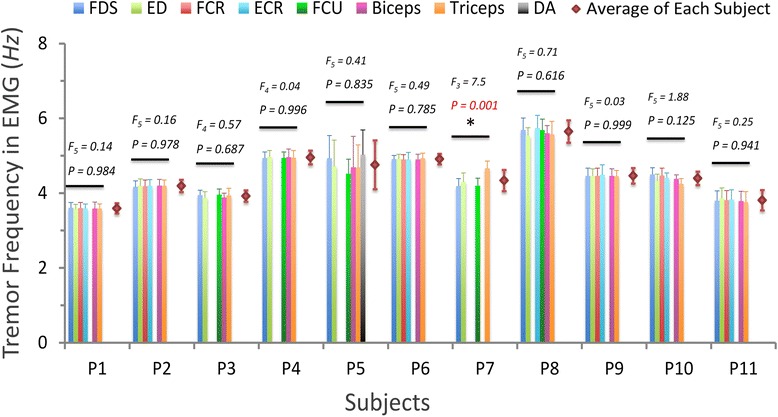
The characteristic tremor frequencies detected from muscle EMGs of 11 PD subjects. For each subject, 6 muscles exhibited significant tremor activities out of the following 8 muscles are recorded: FDS (flexor digitorum superficialis), ED (extensor digitorum), FCR (flexor carpi radialis), ECR (extensor carpi radialis), FCU (flexor carpi ulnaris), Biceps, Triceps, and DA (deltoid anterior). Muscles revealed detectable oscillation frequency are listed as bars (error bars: standard deviation) in this figure, and the resultant F-statistic (F_DF_, in which DF denotes the degrees of freedom) and P-value in the one-way ANOVA of each subject are listed above. The one-way ANOVA test results indicate that there is no significant difference among EMG tremor frequencies of recorded muscles in each subject except P7 (indicated by ‘*’). The subject-specific tremor frequency (red diamond) is averaged from all EMG tremor frequencies of each subject, and the standard deviations of EMG tremor frequencies are given as error bars

We calculated the EMG tremor frequency of all rhythmic muscles in the 11 PD subjects, and the statistics of EMG tremor frequencies are shown in Fig. 4. The rhythmic muscles identified in each subject are listed as color bars, and non-rhythmic muscles (without identifiable frequency) are left blank. The mean value and standard deviation of the EMG tremor frequencies are given to the right side of the bars as diamond markers with error bars. For each subject, one-way ANOVA was applied to test if there were significant differences in EMG tremor frequencies across all rhythmic muscles (Fig. 4). For 10 participating subjects out of 11, there was no significant difference in the tremor frequency among muscles in a subject (the corresponding F-statistic, degrees of freedom, and P value of each subject are given in Fig. 4), indicating that for a specific subject the tremor frequency could be a unique feature of tremor across the muscles in the arm. In subject P7 (*F*_3_ = 7.5, *P* < 0.05), only one muscle (triceps brachii) showed significantly higher tremor frequency compared to other muscles. The characteristic subject specific tremor frequencies of all PD subjects ranged from 3 to 6 *Hz*, which is consistent with previous observations [12, 43, 44].

### Evaluation of degree of inter-muscular synchronization

The degree of inter-muscular synchronization is quantified by 3 estimates in this study: (1) the number of synchronized muscles, (2) the pooled coherence [42], and (3) the pool-averaged coherence. The number of synchronized muscles in each subject is determined by the coherence matrix comprised of all muscle pairs (Table 2), and the pooled coherence and pool-averaged coherence are calculated by pooling all muscle pairs. Figure 3 shows the coherence results for a representative subject (P9). The paired coherence (Fig. 3a) between synchronized muscles shows major peaks at tremor frequency and double tremor frequency, and the coherence level is above the significance level at tremor frequency, but not necessarily at double tremor frequency. The pooled coherence (Fig. 3b) does not show a significant peak at tremor frequency, but instead a discernable peak at double tremor frequency occurs in many subjects (P1, P2, P4, P6, P8, and P9). The pool-averaged coherence (Fig. 3c), on the contrary, displays a quite similar feature as the paired coherence in Fig. 3a, showing significant peaks at both tremor and double tremor frequencies.

**Fig. 5:**
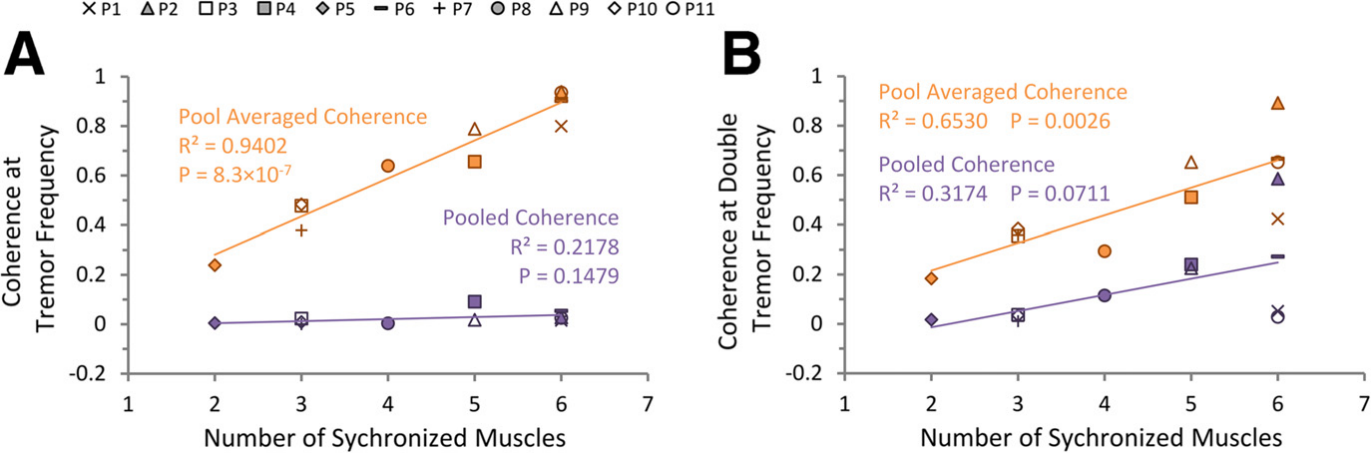
The correlations between different estimates of inter-muscular synchronization. **a** Linear regression between the number of synchronized muscles and the pooled coherence / pool-averaged coherence level at tremor frequency detected from each subject. The pool-averaged coherence at tremor frequency shows fine linear relationship with the number of synchronized muscles, while the pooled coherence doesn’t. **b** Linear regression between the number of synchronized muscles and the pooled coherence / pool-averaged coherence level at double tremor frequency. The pool-averaged coherence at double tremor frequency also shows significant linear relationship with the number of synchronized muscles, while the pooled coherence doesn’t

Comparisons among different estimates of the degree of inter-muscular synchronization are shown in Fig. 5. The number of synchronized muscles is linearly correlated to the pool-averaged coherence level at both tremor frequency (Fig. 5a, orange) and double tremor frequency (Fig. 5b, orange), but not correlated to the pooled coherence level at tremor frequency (Fig. 5a, purple) or double tremor frequency (Fig. 5b, purple). Thus, the number of synchronized muscles and the pool-averaged coherence are two valid estimates for quantifying the degree of inter-muscular synchronization, while the pooled coherence is proven not a sensitive estimate for inter-muscular synchronization.

### Correlation between degree of inter-muscular synchronization and tremor amplitude

Correlation between the inter-muscular synchronization estimates (the number of synchronized muscles, and the pool-averaged coherence) and tremor amplitudes in shoulder and elbow joints was further analyzed. Since the pooled coherence is not a sensitive estimation for the degree of inter-muscular synchronization, it is excluded in this correlation analysis. Tremor amplitudes in shoulder (flexion, abduction and rotation) and elbow (flexion) DOFs of each subject were averaged from all tremor epochs. Subjects P3, P6 and P8 were excluded from this analysis for no detectable tremor in both or either shoulder and elbow joints.

**Fig. 6:**
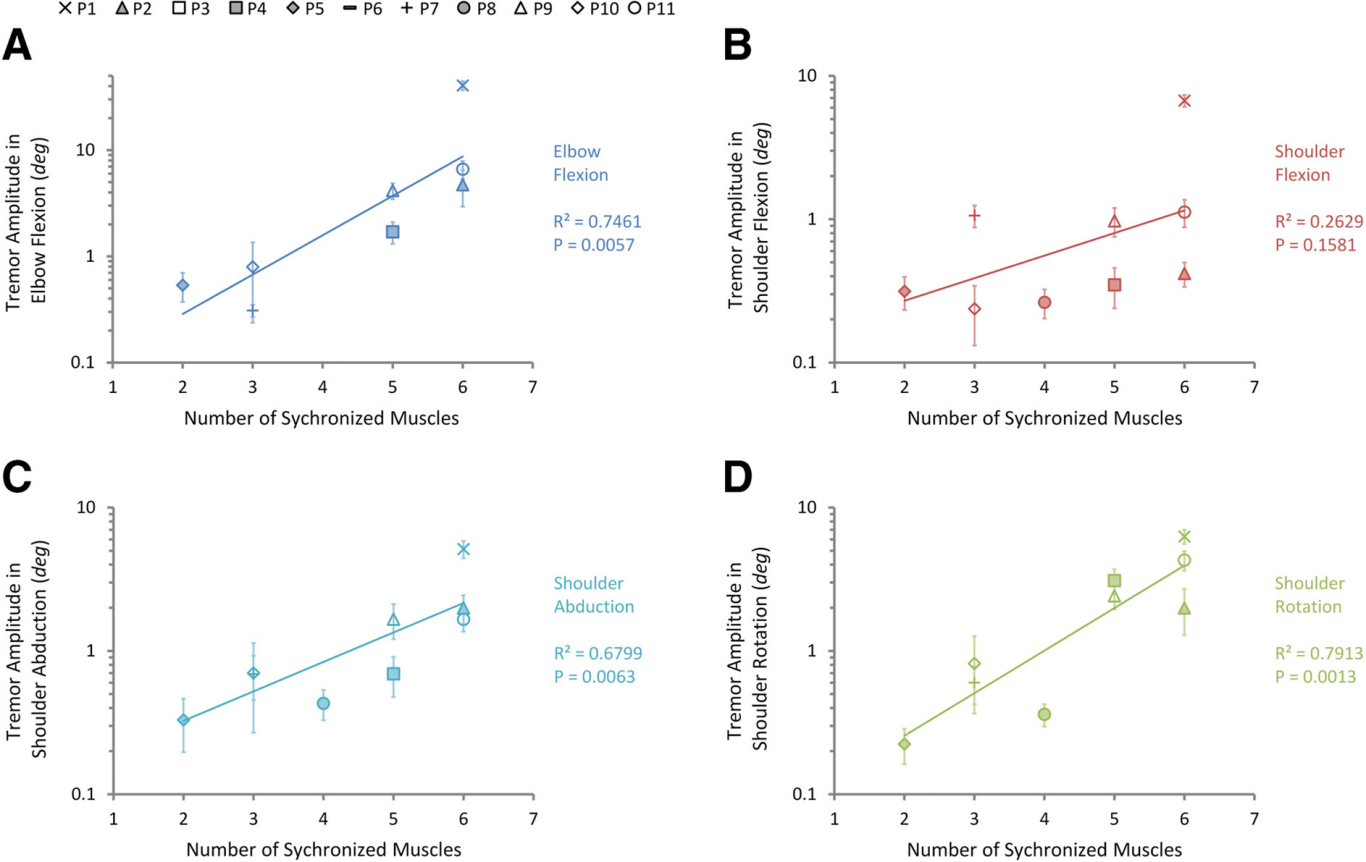
The relationship between the number of synchronized muscles and the tremor amplitudes in shoulder and elbow joints in PD subjects. The correlations between tremor amplitudes in **a** elbow flexion, **b** shoulder flexion, **c** shoulder abduction, and **d** shoulder rotation and the number of synchronized muscles are evaluated by exponential regression. Different subjects are indicated with different markers and the error bars indicate the standard deviation of tremor amplitudes of each subject. The R^2^ (squared correlation coefficients) and P values are given in the figure. Tremor amplitudes in joints are strongly correlated with the number of synchronized muscles except for shoulder flexion that shows only a mild correlation

The relationships between the number of synchronized muscles and tremor amplitudes in joint DOFs are shown in Fig. 6. The number of synchronized muscles is significantly correlated with tremor amplitudes in DOFs of elbow flexion (Fig. 6a), shoulder abduction (Fig. 6c), and shoulder rotation (Fig. 6d) (*P* < 0.05 and R^2^ > 0.5), but only shows a weak correlation with shoulder flexion (Fig. 6b) (*P* = 0.16, R^2^ = 0.26).

**Fig. 7:**
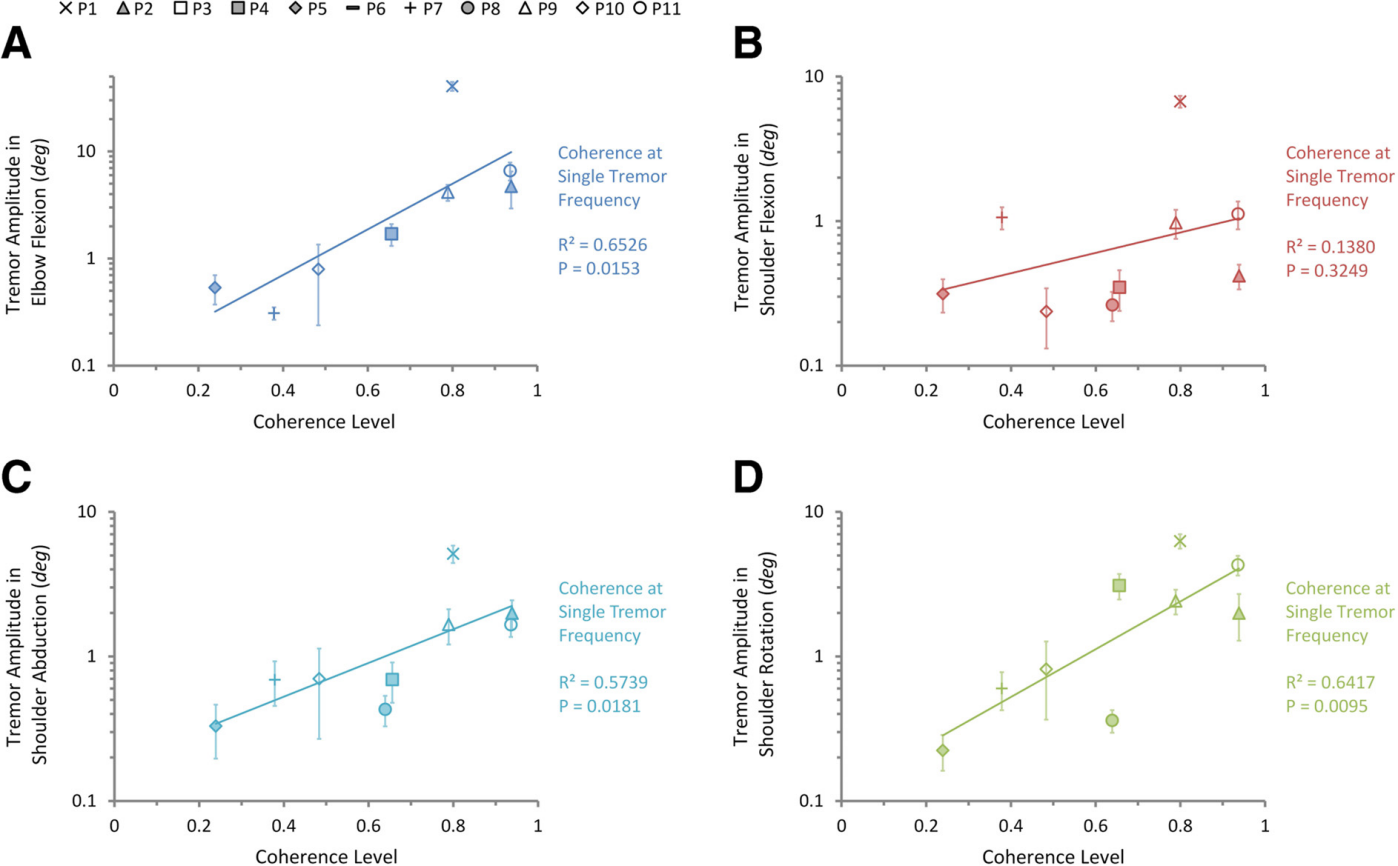
Correlations between the pool-averaged coherence levels at tremor frequency and the tremor amplitudes in shoulder and elbow joints. Exponential regression results show that the tremor amplitudes in DOFs of **a** elbow flexion, **c** shoulder abduction, and **d** shoulder rotation are significantly correlated with the pool-averaged coherence level at tremor frequency, while that of **b** shoulder flexion isn’t. Different subjects are indicated with different markers and the error bars indicate the standard deviation of tremor amplitudes of each subject

**Fig. 8:**
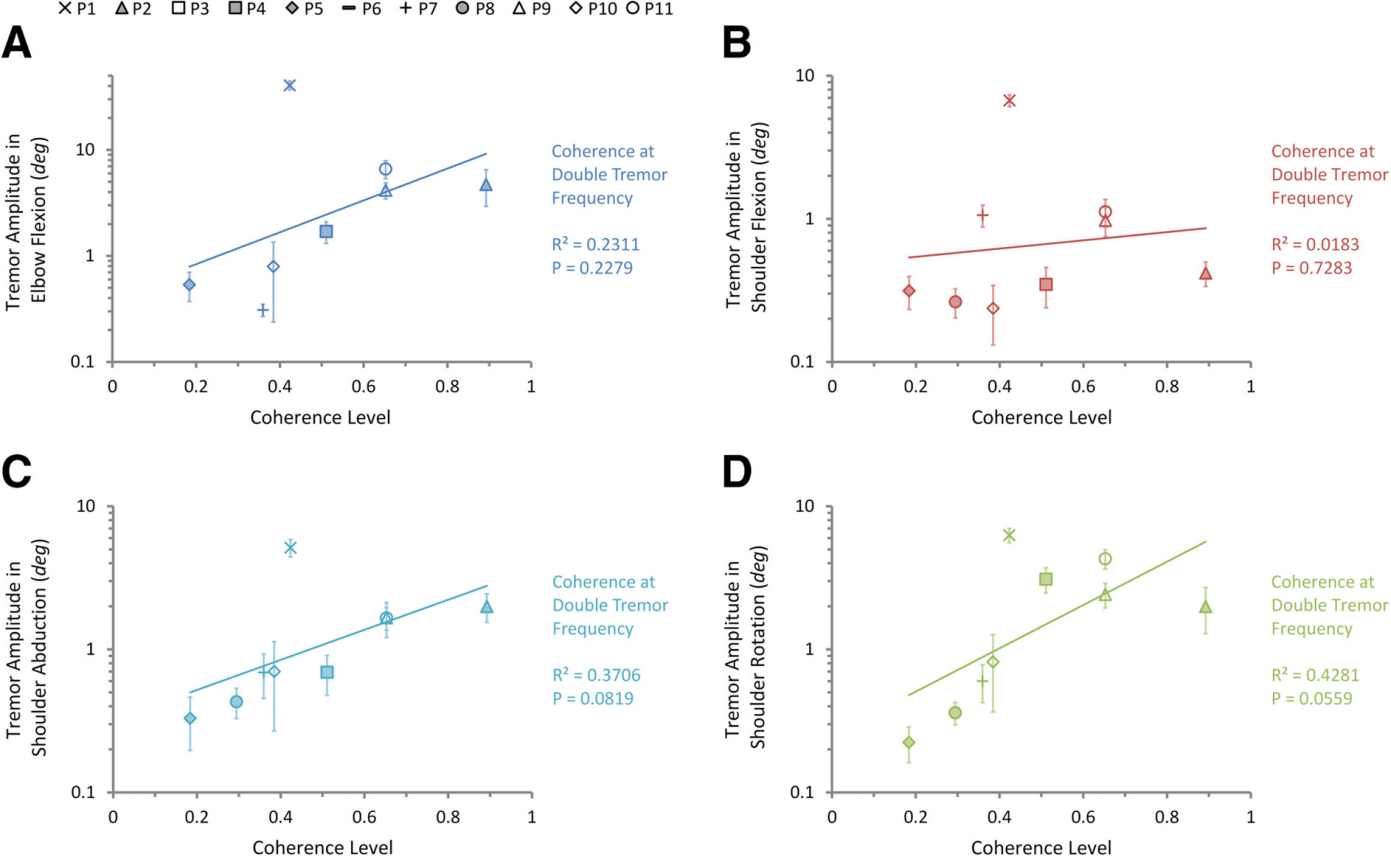
Correlations between the pool-averaged coherence levels at double tremor frequency and the tremor amplitudes in shoulder and elbow joints. Exponential regression results show that the tremor amplitudes in DOFs of **a** elbow flexion, **b** shoulder flexion, **c** shoulder abduction, and **d** shoulder rotation are not significantly correlated with the pool-averaged coherence level at double tremor frequency. Different subjects are indicated with different markers and the error bars indicate the standard deviation of tremor amplitudes of each subject

The relationships between pool-averaged coherence levels and tremor amplitudes in joint DOFs were characterized at tremor frequency and double tremor frequency using linear regression, shown in Figs. 7 and 8 respectively. At tremor frequency, the pool-averaged coherence level is significantly correlated with tremor amplitudes in elbow flexion (Fig. 7a), shoulder abduction (Fig. 7c), and shoulder rotation (Fig. 7d) (*P* < 0.05 and R^2^ > 0.5), but not significantly correlated with tremor amplitude again in shoulder flexion (Fig. 7b) (*P* = 0.32, R^2^ = 0.14). At double tremor frequency, the correlation is weak (*P* > 0.05, R^2^ < 0.5) (Fig. 8). The strong correlation at tremor frequency for the pool-averaged coherence is consistent with the fact that paired coherence between muscles displays a stronger component at tremor frequency than at double tremor frequency. Thus, they are sensitive measurements for quantifying the level of inter-muscular synchronization.

## Discussion

In this study, EMGs of six upper extremity muscles recorded along with resting tremor in 11 tremor-dominant PD patients were analyzed to investigate how these muscles are synchronized to act in modulating tremor activity at joints. The synchronization appears to happen at intra-muscular and inter-muscular levels. A subject-specific tremor frequency was identifiable from each PD subject except for P7, indicating that the bursts in EMGs of the arm muscles in each subject shared the same characteristic tremor frequency (Fig. 4). However, the degree of inter-muscular synchronization varied in different subjects (Fig. 5), and only a subset of the recorded muscles in a subject displayed significant correlation among them (Table 2). This result provides neuromechanical evidence that the degree of inter-muscular synchronization is related to tremor intensity.

We further examined this phenomenon by correlation analysis between estimates of synchronization level and tremor amplitudes in the shoulder and elbow joints. The amplitude of joint tremor is positively correlated to the number of synchronized muscles in each subject (Fig. 6), as well as the pool-averaged coherence at tremor frequency (Fig. 7). These results suggest that the presence of rhythmic firings in muscle EMG is not sufficient to produce prominent tremor activity in peripheral joints. Synchronization of these rhythmic firings among a group of muscles in one limb is the driving force that contributes to modulating the tremor intensity.

**Fig. 9:**
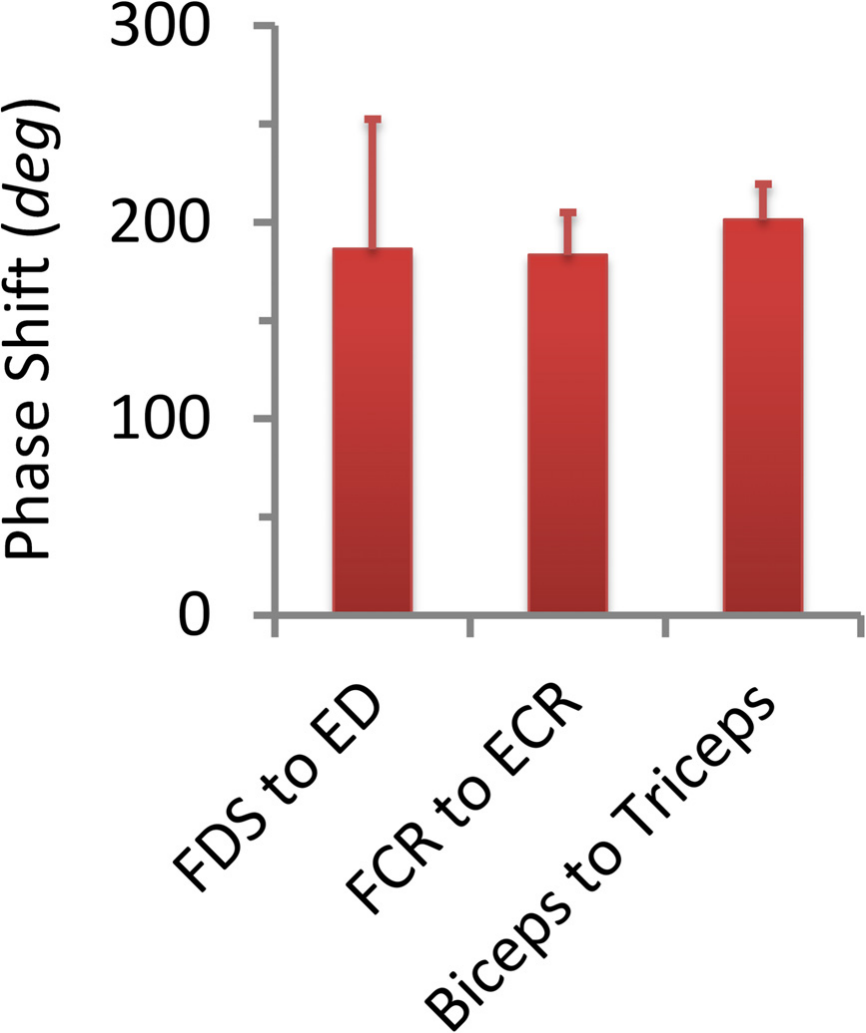
The phase shift between 3 pairs of antagonistic muscles averaged from all PD subjects. The error bars indicate the standard deviation of phase shift calculated from all subjects

One interesting finding in this study is that the synchronization between muscles at tremor frequency is always stronger than that at double tremor frequency (Fig. 5). Furthermore, the pool-averaged coherence at tremor frequency (Fig. 7) is more strongly correlated to tremor amplitude than that at double tremor frequency (Fig. 8). Thus muscles are inter-coupled more strongly at tremor frequency than at double tremor frequency in the periphery. This is in contrast to the finding that the oscillations in cortical and sub-cortical areas showed a more preeminent coupling at double tremor frequency [14]. It suggests that between the two central driving signals oscillating at single tremor and double tremor frequencies, the signal at single tremor frequency tends to synchronize the muscles, while the signal at double tremor frequency provides a direct drive to the muscles [14]. The results further imply that a change in frequency content has taken place during corticospinal transmission of tremor signals. Hao et al. [27] proposed a corticospinal mechanism of tremor signal transmission, and described the process within the propriospinal neuron (PN) network, where an alternating pattern of antagonistic muscle activation is generated from the pair of central oscillation signals. The central oscillation at double tremor frequency is gated at the PN network by the signal with tremor frequency to produce alternating bursts at tremor frequency that drive a pair of antagonistic muscles. This PN processing consequently converts the central tremor signal with dominant double tremor frequency to single tremor frequency in the peripheral muscles, and translates frequency contents to give rise to a more favorable condition for tremor to occur in the periphery. This favorable condition is manifested in the near half-cycle phase shift between synchronized antagonistic muscles [12, 43, 44], which was 191.6° ± 40.9° in the subjects (Fig. 9).

Three different measures were used in this study for evaluating the degree of inter-muscular synchronization. We were interested in finding a single index that can correlate to tremor amplitude. These measures were evaluated for their ability to characterize the synchronization of a group of muscles. The number of synchronized muscles and the pool-averaged coherence were found to be both sensitive estimates of inter-muscular synchronization (Figs. 6 and 7), while the pooled coherence did not demonstrate a significant correlation with inter-muscular synchronization (Fig. 5). These results indicate that the paired and pool-averaged coherences indeed are better representations for inter-muscular synchronization. Between the two measures, we prefer pool-averaged coherence for its simplicity and convenience in calculation. The number of synchronized muscles is determined by identifying the subgroup of muscles that demonstrated significant coherence between all combinations in the paired coherence analysis. A possible deficit of this estimate is that the determination of the significance level of coherence is based on the choice of the confidence level, and this may influence the outcome of the number of synchronized muscles. The pooled coherence was used in the evaluation of motor-unit synchronization within a muscle during physiological tremor [42, 45]. However, it is shown in this study that it is not a sensitive estimate for inter-muscular synchronization in PD patients. This is because in the calculation of pooled coherence, the “pooling” of cross-spectra among muscles takes into account the phase of the spectra (Eq. 4), and the cross-spectra at tremor frequency for antagonistic muscles tend to cancel with each other due to a phase shift of about 180° (Fig. 9), thereby reducing the pooled coherence to an insignificant level at tremor frequency (Fig. 3b). We proposed the pool-averaged coherence as an estimate for inter-muscular synchronization in a group of muscles in PD patients. The pool-averaged coherence is defined as the weighted sum of magnitude squared coherence of all muscles in the pool, and removes phase information from the cross spectra (Eq. 6). The pool-averaged coherence at tremor frequency and double tremor frequency is linearly correlated to the number of synchronized muscles (Fig. 5), confirming that the pool-averaged coherence is a sensitive measure for inter-muscular synchronization. However, the pool-averaged coherence at tremor frequency shows a stronger correlation with tremor amplitude at joints than that at double tremor frequency (Figs. 7 and 8). Thus, the pool-averaged coherence yields a consistent estimate with the number of synchronized muscles, but without the influence of subjective choice for a significance level.

## Conclusion

In this study, the neuromechanical coupling among a set of muscles in the upper extremity is assessed by coherence analysis of recorded EMG signals during resting tremor in 11 tremor-dominant PD subjects. The main findings in this study are summarized as follows. (1) Almost all muscles in the arm share the same characteristic subject-specific tremor frequency in a subject. (2) Only a subset of the spontaneously firing muscles is synchronized in modulating tremor intensity; and the degree of inter-muscular synchronization is positively correlated with tremor amplitudes at joints. (3) The number of synchronized muscles and the pool-averaged coherence are sensitive estimates for inter-muscular synchronization.
